# Analgesic Efficacy of *Curcuma longa* (Curcumin) after Surgical Periodontal Therapy

**DOI:** 10.3290/j.ohpd.b2572979

**Published:** 2022-01-20

**Authors:** Mansour Al-Askar, Abdulrahman M AlMubarak, Montaser N. Alqutub, Sameeer Mokeem, Fawad Javed, Fahim Vohra, Tariq Abduljabbar

**Affiliations:** a Associate Professor, Department of Periodontics and Community Dentistry, King Saud University, Riyadh, Saudi Arabia. Statistical analysis, investigation, methodology, validation, visualisation, reviewed and approved final version.; b Assistant Professor, Department of Periodontics and Community Dentistry, King Saud University, Riyadh, Saudi Arabia. Data curation, formal analysis, reviewed and approved final version.; c Associate Professor, Department of Periodontics and Community Dentistry, King Saud University, Riyadh, Saudi Arabia. Investigation, methodology, validation, visualisation, reviewed and approved final version.; d Professor, Department of Periodontics and Community Dentistry, King Saud University, Riyadh, Saudi Arabia. Data curation, formal analysis, reviewed and approved final version.; e Assistant Professor, Department of Orthodontics and Dentofacial Orthopedics, Eastman Institute for Oral Health, University of Rochester, Rochester, NY, USA. Reviewed and approved final version.; f Professor, Department of Prosthetic Dental-Sciences, College of Dentistry, King Saud University, Saudi Arabia. reviewed and approved final version. Data curation, investigation, methodology, validation, drafted initial manuscript.; g Professor, Department of Prosthetic Dental Sciences, College of Dentistry, King Saud University, Saudi Arabia. Conceptualization, data curation, formal analysis, funding acquisition, investigation, methodology, project administration, supervision, validation, visualisation, drafted initial manuscript, reviewed and approved final version.

**Keywords:** curcumin, mefenamic acid, pain, periodontal flap surgery

## Abstract

**Purpose:**

To compare the analgesic efficacy of orally administered *Curcuma longa* (curcumin) and mefenamic acid (MA) after surgical periodontal therapy (SPT).

**Materials and Methods:**

Seventy-six periodontitis patients were randomly divided into two groups. In the test group, patients received curcumin capsules (200 mg), and in the control group, patients received MA (500 mg). All patients underwent post-operative antibiotic therapy using 500 mg amoxicillin and 400 mg metronidazole for 7 days. Post-operative pain and discomfort were evaluated using the numerical rating scale (NRS) and verbal rating scale (VRS), respectively. Evaluation were performed after 24 (T1), 48 (T2), and 72 h (T3). Group comparisons were done using Student’s t-test and the Mann-Whitney U-test. The level of statistical significance was established at p < 0.05.

**Results:**

All patients had stage 3/grade C periodontitis. The mean age of individuals in the test and control groups were 58.4 ± 7.3 and 57.2 ± 5.2 years, respectively. A family history of periodontal diseases was reported by 37.5% and 47.4% individuals in the test and control groups, respectively. In the test and control groups, the total mean duration of periodontal surgery was 168.2 ± 12.2 and 173.4 ± 10.7 min, respectively. There was no statistically significant difference in the mean NRS and VRS scores among patients in the test and control groups. In both groups, there was no statistically significant difference in the change in NRS scores at any time point.

**Conclusions:**

Compared with MA, curcumin is ineffective for pain and discomfort management after SPT. The possibility of the results being biased due to lack of operator blinding cannot be overlooked.

Post-operative pain after oral surgical interventions such as tooth extraction and periodontal flap procedures is often disquieting for patients.^33,34^ Traditionally, non-steroidal anti-inflammatory drugs (NSAIDs) such as diclofenac, mefenamic acid (MA), acetyl salicylic acid and propionic acid derivatives are prescribed to patients for the management of post-operative pain.^16,20^ The NSAIDs inhibit cyclooxygenase-2 (COX-2), thereby blocking production of prostaglandins,^12^ and are beneficial for the management of mild to moderate levels of pain.^18^ However, the analgesic efficacy of NSAIDs is often compromised in patients experiencing severe pain.^44^ The NSAIDs should not be prescribed to patients using anticoagulants such as warfarin and clopidogrel,^7^ and their potential side-effects include gastrointestinal- and hepatotoxicity.^7,9^ The global use of herbal medications for pain relief has increased over the years;^2,30,41^ their consumers often perceive that medications derived from medicinal plants are safe and have no undesirable side-effects compared with synthetic pharmacological drugs.^6,29^

Curcumin is an organic polyphenol present in the roots of *Curcuma* species, such as turmeric.^3^ It adjusts the inflammatory response by down-regulating the activity of lipoxygenase and COX-2. Moreover, curcumin retards the production of inflammatory cytokines such as interleukin (IL)-1, IL-6, IL-12 and tumor necrosis factor-alpha (TNF-α).^28^ It has also been reported that curcumin is a potent anti-oxidant and anti-inflammatory agent,^35^ and is claimed to relieve pain, e.g. arthritis, burn pain and neuropathic pain.^43^ In a randomised controlled clinical trial, the effectiveness of curcumin (test group) was compared with MA (control group) for managing post-operative pain after surgical extraction of impacted third molars.^33^ In that study, self-perceived post-operative pain was assessed using the numerical rating scale (NRS). The results showed that although patients in both groups perceived less pain than baseline pain levels, individuals in the test group perceived significantly less post-operative pain compared with the control group. The authors concluded that curcumin is useful in the management of pain following surgical extraction of impacted wisdom teeth.^33^ A limited number of clinical studies have assessed the analgesic efficacy of a curcumin-based gel following periodontal flap surgical procedures.^5,34^ Their results showed that curcumin-containing mucoadhesive films are useful in reducing postoperative pain and swelling following periodontal surgical interventions.^5,34^ A thorough review of the relevant literature yielded no studies which compared the analgesic efficacy of orally administered *Curcuma longa* (curcumin) and MA after surgical periodontal therapy (SPT). Thus, the aim of this study was to compare the analgesic efficacy of curcumin and MA after SPT. The authors hypothesise that compared with MA, curcumin is not effective for pain management after SPT.

## Materials and Methods

### Ethics Statement

The present study was performed following guidelines recognized by the Declaration of Helsinki as revised in 2013 for experimentation involving human patients. All volunteer participants were requested to read and sign a consent form written in simple English and Arabic. All participants were informed that they could withdraw at any phase of the study without consequences. Ethical approval was obtained from the research ethics committee of the Centre for Specialist Dental Practice and Clinical Research (UDCRC/025-16).

### Eligibility Criteria

Patients with periodontitis were included. Self-reported smokers,^25^ habitual consumers of alcohol^19^ and patients with systemic illnesses, including but not limited to self-reported oral and systemic cancer, HIV/AIDS, diabetes mellitus (DM), prediabetes, hepatic and renal diseases, and cardiovascular diseases, were excluded.^8,22,27,39^ Moreover, overweight and obese individuals, pregnant and/or lactating females, and patients who had consumed probiotics, corticosteroids/steroids, or bisphosphonates within the past 90 days were excluded. Likewise, subjects who were undergoing dental prophylaxis or any form of oral or craniofacial surgery were also eliminated.

### Preoperative Staging and Grading of Periodontitis

Patients diagnosed with periodontitis were included. Periodontitis was defined using the following parameters: horizontal marginal bone loss (MBL), probing depth (PD) of ≥ 4 mm, and clinical attachment loss (AL) of at least 1–2 mm.^38^ Pre-operative patient records were evaluated to determine the staging and grading of periodontitis by one trained investigator (Kappa 0.89).

### Design, Randomisation, and Allocation Concealment

A parallel-arm trial design as described elsewhere^37^ was applied in the current investigation. For randomisation, a computer program (www.random.org, Randomness and Integrity Services; Dublin, Ireland) was used, and allocation to the study-groups was done via block randomisation.^11^

### Surgical Procedure

All surgical procedures were performed in one session by a trained and experienced periodontist. Under local anesthesia, all patients underwent full-mouth dental prophylaxis using an ultrasonic hand scaler (Woodpecker Uds-J Ultrasonic Scaler, EMS Compatible Original; Guangzhou, China). In both arches, gingival sulcular incisions were maed around the affected teeth using a sterile No. 15 surgical blade (iSmile Dental Products, Prehma #15 sterile stainless steel surgical scalpel blade; Sacramento, CA, USA), and buccal and lingual/palatal flaps were elevated using sterile periosteal elevators (Syze UK, DS-1004; London, UK) as described elsewhere.^14^ Mechanical curettage of teeth and root surfaces was performed using sterile curettes (Hu-Friedy; Chicago, IL, USA) to remove subgingival plaque, calculus, diseased granulation tissue and pocket epithelium. The flaps were repositioned and sutured using 3-0 interrupted sutures (Unify PGA Surgical Sutures, Henry Schein; Berlin, Germany). The duration of the surgical intervention was also retrieved from patients’ dental records.

### Grouping

The principal investigator communicated with participants of the assigned treatment group. Post-operatively, participants were divided into 2 groups. 1. Test group: patients received curcumin capsules (200 mg) (Terry Natural Products, CuraMed; Green Bay, WI, USA); 2. control group: patients received one tablet of MA (500 mg). In both groups, participants were prescribed antibiotics (amoxicillin 500 mg and metronidazole 400 mg) 3x daily (every 8 h) for 7 days. In the present trial, the dosage of curcumin was based on results from previous reviews.^15,28^ The participants in the test and control groups were advised to orally take 1 MA tablet and 2 curcumin capsules, respectively, immediately after the procedure and then every 8 h for 3 days. After the third day, participants in the test and control groups were advised to take the respective analgesics as needed for pain.

### Questionnaire, Evaluation of Post-operative Pain and Discomfort Scores, Blinding

A standardised questionnaire was given to all participants to collect information regarding age, gender, family history of periodontal diseases, drug allergies, and intensity of post-operative pain. The questionnaire was administered to all participants by a trained investigator (SM). In both groups, evaluation of post-operative pain was performed using the numerical rating scale (NRS) and discomfort using a 4-point verbal rating scale (VRS) (1 = none; 2 = mild; 3 = moderate; 4 = severe).^5,42^ Three pain evaluations were performed by phone calls with the patients after 24 (T1), 48 (T2), and 72 h post-operatively. This procedure was performed by one trained and calibrated investigator (Kappa 0.92) who was blinded to the study groups.

### Statistical Analysis

Statistical analysis was conducted using a software program (SPSS v.18, IBM; Chicago, IL, USA). Group comparisons were performed using Student’s t-test and the Mann-Whitney U-test. Power analysis was calculated based on results obtained from a pilot investigation using a software program (G*Power version 3.1.5., University of Kiel; Kiel, Germany). A t-test of independent means (test and control groups) was set as the statistical test to perform power analysis using an effect size of 1 and alpha of 5%. Bonferroni’s post-hoc adjustment was used for multiple comparisons. It was estimated that inclusion of at least 38 individuals per group was necessary to attain a study power of 91.5%. p-values below 0.05 were considere statistically significant.

## Results

### Recruitment of Study Participants

**Fig 1:**
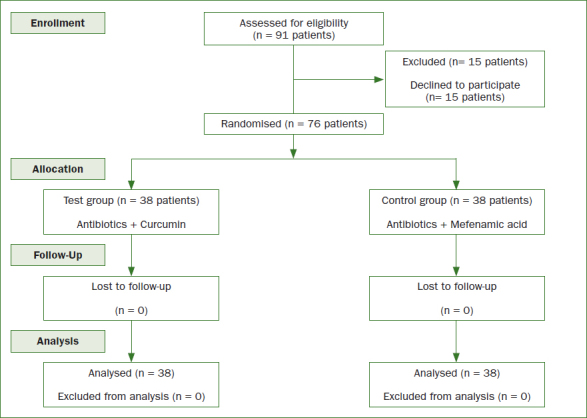
CONSORT flow diagram for patient recruitment.

Ninety-one individuals were initially invited to the present study. Fifteen individuals (11 females and 4 males) refused to participate in the present study. These individuals (n = 15) refused to sign the written informed consent form. In total, 76 individuals (38 per group) were included (Fig 1).

### Characteristics of the Study Cohort

In the test and control groups, 26 and 28 individuals, respectively, were males. The mean ages of individuals in the test group was 58.4 ± 7.3 and 57.2 ± 5.2 years in the control group. There was no statistically significant difference in the mean age of males and females in the test and control groups. A family history of periodontal diseases was reported by 37.5% in the test group and 47.4% in the control group. None of the patients reported drug allergies (Table 1). There was no statistically significant difference in the preoperative periodontal inflammatory parameters in both groups (Table 1). As per the periodontal diagnostic records, all patients had stage 3/grade C periodontitis. In the test and control groups, the total mean duration of periodontal surgery was 168.2 ± 12.2 min in the test group and 173.4 ± 10.7 min in the control group. None of the participants in the test and control group reported allergic reactions or complications associated with their prescription medication.

**Table 1:** Characteristics of the study groups

Parameters	Test group	Control group
Number of patients	38	38
Gender (male:female)	26:12	28:10
Age in years (all patients)	58.4 ± 7.3 years	57.2 ± 5.2 years
Age of males in years	61.2 ± 4.3 years	62.4 ± 5.1 years
Age of females in years	56.1 ± 2.9 years	55.7 ± 4.1 years
**Periodontal parameters**		
Plaque index	2.7 ± 0.3	2.8 ± 0.2
Gingival index	3.02 ± 0.2	2.9 ± 0.05
Probing depth	4.2 ± 0.07 mm	4.3 ± 0.02 mm
Clinical attachment loss	6.1 ± 0.2 mm	5.9 ± 0.03 mm
Marginal bone loss (mesial surface)	4.7 ± 0.06 mm	4.6 ± 0.03 mm
Marginal bone loss (distal surface)	4.6 ± 0.04 mm	4.5 ± 0.05 mm
Number of missing teeth	16.2 ± 2.5 teeth	16.6 ± 1.6 teeth
**Family history of periodontal diseases**		
Yes	15 (37.5%)	18 (47.4%)
No	5 (13.2%)	10 (26.3%)
I don’t know	15 (49.3%)	10 (26.3%)
Allergies to antibiotics (penicillin)	None	None
Allergies to NSAIDs	None	None

NSAIDs: non-steroidal anti-inflammatory drugs.

### Numerical Rating Scale and Verbal Rating Scale Scores

**Fig 2:**
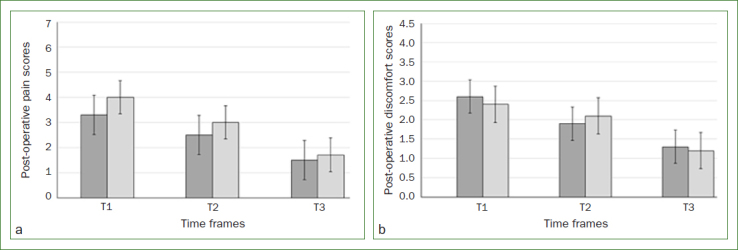
a. Mean numerical rating scale scores in the test (dark grey bars) and control groups (light grey bars) after 24 h (T1), 48 h (T2) and 72 h (T3); b. Mean verbal rating scores in the test (dark grey bars) and control groups (light grey bars) after 24 h (T1), 48 h (T2) and 72 h (T3).

The mean NRS and VRS scores assessed at different post-operative time points (T1, T2 and T3) were comparable (p > 0.05) among patients in the test and control groups (Figs 2a and 2b). There was no statistically significant difference in the mean NRS and VRS scores assessed at the 3 different post-operative time points among males and females in either group (data not shown).

### Change in Numerical Rating Scale and Verbal Rating Scale Scores at Different Time Intervals

In the test and control groups, there was no statistically significant change in the NRS scores assessed at the 3 different post-operative time points (Tables 2 and 3). There was no statistically significant difference in the change in mean NRS and VRS scores assessed at any of the post-operative time points among males and females in either group (data not shown).

**Table 2:** Comparison of change in numerical rating scale scores in the test and control groups at different time intervals

Time interval	Groups	Change in mean NRS score	p-value
T1 vs T2	Test group	0.8	0.13
	Control group	1	
T1 vs T3	Test group	1.8	0.06
	Control group	2.3	
T2 vs T3	Test group	1	0.15
	Control group	1.3	

NRS: numerical rating scale; T1: 24 h after periodontal surgery; T2: 48 h after periodontal surgery; T3: 72 h after periodontal surgery.

**Table 3:** Comparison of change in verbal rating scale scores in the test and control groups at different time intervals

Time interval	Groups	Change in mean VRS score	p-value
T1 vs T2	Test group	0.7	0.15
	Control group	0.3	
T1 vs T3	Test group	1.3	0.22
	Control group	1.2	
T2 vs T3	Test group	0.6	0.21
	Control group	0.9	

VRS: verbal rating scale; T1: 24 h after periodontal surgery; T2: 48 h after periodontal surgery; T3: 72 h after periodontal surgery.

## Discussion

In the present study, stringent eligibility criteria, such as exclusion of self-reported tobacco-product users and immunosuppressed patients, were adopted. It is well known that tobacco smoking and systemic diseases such as poorly-controlled DM and habits such as cigarette smoking are risk factors for periodontitis.^24,27^ Although patients included in the present investigation were presumably systemically healthy and were non-smokers, it is imperative to interpret this information with caution, as laboratory-based investigations such as assessment of serum glycemic and salivary cotinine levels were not assessed. However, other factors, e.g. as advancing age, compromised routine oral hygiene maintenance, low socioeconomic status and low educational level, are also associated with the occurrence and progression of periodontitis.^10,27,31^ According to Lertpimonchai et al,^31^ the risk of periodontitis is nearly 5 times higher in patients with a poor oral hygiene. Such factors may have contributed to the progression of periodontitis in the patient population assessed.

The present results showed no statistically significant difference in the postoperative NRS and VAS scores in the test and control groups. It is therefore tempting to speculate that curcumin and MA are equally effective in reducing self-perceived pain after oral surgical interventions. However, it is important to cautiously interpret such a conclusion, as a number of factors may have biased the results. Firstly, the principal investigator was aware of the medications prescribed to patients in the test and control groups. Although the study was power adjusted, the possibility of bias cannot be overlooked. One justification for this is that patients using curcumin were advised to use 2 capsules whereas those in the control group were taking one tablet of MA. In this regard, patients were possibly aware of which group they belonged to. Furthermore, from an ethical standpoint, the authors could not conceal the objectives of the present study from the participants. Furthermore, to the authors’ knowledge from indexed literature, a consensus has yet to be reached regarding the oral dosage of curcumin that is most effective for the management of post-operative pain and discomfort following oral surgical interventions. In this context, the authors relied upon data from indexed literature to estimate the most effective dosage of curcumin. Results of a clinical trial showed that in contrast to NSAIDs, curcumin demonstrates superior post-surgical anti-inflammatory effect when orally consumed at a dose of 1.2 g daily for 6 days.^21^ Nevertheless, it is difficult to predict the precise role of curcumin or MA in reducing post-operative discomfort or pain as all participants were prescribed antibiotics. From a biomedical ethics perspective, the authors were unable to include additional treatment groups (patients prescribed curcumin or MA only). This was done to minimise the risk of post-surgical infections that could have otherwise influenced pain and discomfort scores after the procedure. In the present study, none of the participants reported any side-effects after consuming any of the prescribed medications. These results suggest that curcumin is a safe herbal medication for the management of post-operative pain and discomfort. This is supported by the results of a phase-I clinical trial in which oral intake of curcumin at doses up to 8 g/day induced no side-effects or complications.^13^ However, in order to declare curcumin a reliable replacement for traditional NSAIDs warrants further studies.

## Conclusion

Compared with MA, curcumin is ineffective for pain and discomfort management after surgical periodontal debridement in patients with periodontitis. The possibility of the results being biased due to lack of operator blinding cannot be overlooked.
